# Expression of calpain-calpastatin system (CCS) member proteins in human lymphocytes of young and elderly individuals; pilot baseline data for the CALPACENT project

**DOI:** 10.1186/1742-4933-10-27

**Published:** 2013-07-08

**Authors:** Anna Mikosik, Jerzy Foerster, Aleksandra Jasiulewicz, Joanna Frąckowiak, Giuseppina Colonna-Romano, Matteo Bulati, Silvio Buffa, Adriana Martorana, Calogero Caruso, Ewa Bryl, Jacek M Witkowski

**Affiliations:** 1 Department of Pathophysiology, Medical University of Gdańsk, Gdańsk, Poland; 2 Department of Social and Clinical Gerontology, Medical University of Gdańsk, Gdańsk 7, Poland; 3 Department of Biopathology and Medical and Forensic Biotechnologies (DIBIMEF), University of Palermo, Palermo, Italy

**Keywords:** Ageing, μ-Calpain, m-calpain, Calpastatin, Human, Lymphocytes, Quantitative flow cytometry

## Abstract

**Background:**

Ubiquitous system of regulatory, calcium-dependent, cytoplasmic proteases – calpains – and their endogenous inhibitor – calpastatin – is implicated in the proteolytic regulation of activation, proliferation, and apoptosis of many cell types. However, it has not been thoroughly studied in resting and activated human lymphocytes yet, especially in relation to the subjects’ ageing process. The CALPACENT project is an international (Polish-Italian) project aiming at verifying the hypothesis of the role of calpains in the function of peripheral blood immune cells of Polish (Pomeranian) and Italian (Sicilian) centenarians, apparently relatively preserved in comparison to the general elderly population. In this preliminary report we aimed at establishing and comparing the baseline levels of expression of μ- and m-calpain and calpastatin in various, phenotypically defined, populations of human peripheral blood lymphocytes for healthy elderly Sicilians and Poles, as compared to these values observed in young cohort.

**Results:**

We have found significant differences in the expression of both μ- and m-calpain as well as calpastatin between various populations of peripheral blood lymphocytes (CD4+, CD8+ and CD19^+^), both between the age groups compared and within them. Interestingly, significantly higher amounts of μ- and m-calpains but not of calpastatin could be demonstrated in the CD4^+^CD28- and CD8^+^CD28^-^ lymphocytes of old subjects (but not in the cells of young individuals), as compared to their CD28^+^ counterparts. Finally, decreased expression of both calpains in the elderly T cells is not related to the accumulation of effector/memory (CD45RO^+^) cells in the latter, as the expression of both calpains does not differ significantly between the naïve and memory T cells, while is significantly lower for elderly lymphocytes if both populations are taken separately.

**Conclusions:**

Observed differences in the amounts of CCS member proteins between various populations of lymphocytes of young and elderly subjects may participate in the impaired proliferative activity of these cells in the elderly.

## Background

The ubiquitous, cytoplasmatic calpain-calpastatin system (CCS) consists of a group of cysteine proteases known as calpains and of their endogenous inhibitor - calpastatin. Calpains are extremely calcium-dependent, as their proteolytic activity requires a sufficiently high concentration of calcium ions, usually much higher than that observed in the cytoplasm of resting cells [1,2]. Calpain family consists of a classic pair of the enzymes (μ-calpain and m-calpain-see below) that do not exhibit tissue specificity and are therefore referred to as the ‘typical’ and ‘ubiquitous’, and a larger group of tissue - specific calpains, occurring only in certain organs. Their respective names - μ-calpain (calpain I) and m-calpain (calpain II) - reflect the respective in vitro Ca2^+^ concentrations required for full proteolytic activity; μ-calpain respectively requires 1–100 μM Ca^2+^ and m-calpain 0.1 - 1 mM Ca^2+^[3]. On the other hand, calpastatin is the only natural, endogenous, and first of all specific inhibitor of calpains, coexisting with the enzymes and exerting no significant influence on other enzymes [3-5]. Together, the two ubiquitous calpains and calpastatin, remaining in a precarious balance within cytoplasm, form the CCS system.

Physiological relevance of calpains has already been recognized. Their importance for cellular physiology stems from their already known substrate list on one side and their modus operandi on the other. Thus, literally hundreds of cellular proteins from virtually all functional families had been shown to undergo calpain-dependent cleavage; yet, although calpains can completely hydrolyze some of their substrates, against most of them they do not operate like a normal protease, but by limited hydrolysis of only one or a few sites in the substrate molecule they modulate its activity and function [1,6,7]. This “biomodulation” of calpain substrates (controlled by internal balance within the CCS on one side, and by the momentous levels of cytoplasmic [Ca2^+^] on the other) can affect the generation and transduction of signals leading to cell proliferation, functional activation or apoptosis [8-10]. CCS system activity has already been implicated in the pathologies ranging from Alzheimer’s disease to muscular dystrophies to multiple sclerosis to cancers [10-13]. Its recognized physiological roles make it a potentially important regulator of the immune response.

We have recently demonstrated that calpain activity is increased in the B-CLL leukemic cells [14], in the CD4^+^ T cells of rheumatoid arthritis patients [15] and in acute childhood leukemia blasts (in preparation). We also have preliminarily data suggesting the same for CD4^+^ lymphocytes of healthy elderly. Thus it is feasible that the system may be involved (beneficially or otherwise) in the functioning of T lymphocytes and its deregulation or disruption may impact on or participate in more general way in the process of immune system ageing, affecting also other populations of the immune cells. In the available literature there are no data on the relative abundance or activity of the CCS members in human blood lymphocytes in relation to ageing.

Centenarians (technically people whose lifespan extended beyond 95 years of age) are thought to be a (genetically? environmentally?) selected group of human beings ageing extremely successfully [16,17]. Their successful ageing implies also relatively good state of their immune systems. As the latter heavily depend on the timely and balanced activation, proliferation, secretion and ultimately apoptotic death of many interrelated and cooperating subpopulations of the adaptive immune system, we proposed a hypothesis of different activity of the CCS in the immune cells of centenarians (as compared to younger healthy elderly and to the healthy young cohort). Study of this hypothesis is the goal of the Polish-Italian CALPACENT project. In more detail, its overall aim is to compare the relative abundances, activities and roles of CCS member proteins in various peripheral blood lymphocyte populations from Polish (Pomeranian) and Italian (Sicilian) centenarians with those observed in elderly and young cohorts. This preliminary report provides the baseline values of expression of the CCS system members in major T and B lymphocytes populations of young and elderly cells, as a starting point for its more detailed characteristics.

## Methods

A total of 26 healthy individuals (proportionally from Poland and Italy) were included in the study, divided into two groups: young (4 men and 6 women, age range: 26–38, median 29,5 years) and old (6 men and 10 women, age range: 76–95, median 86,5 years). All subjects were in good health according to their clinical history and none of them had infectious, inflammatory, neoplastic or autoimmune diseases at the time of the study. The study was approved by both Polish and Italian committees for ethics in biomedical research at the partners’ institutions; the subjects were informed about the purpose of the study and about their own involvement and gave their consent.

Whole blood samples were obtained in Vacutainer™ tubes containing ethylenediamine-tetraacetic acid (EDTA). Peripheral blood mononuclear cells (PBMC) were isolated by density gradient centrifugation on Lympholyte^®^ (Cedarlane, Canada) and directly immunostained with antibody conjugates detecting the following lymphocyte antigens: CD4-PE-Cy5, CD8-PE-Cy5 (DAKO, Denmark) and CD28-PE, CD45RA-PE, CD45RO-PE-CF594 (Becton–Dickinson, USA) - for T lymphocytes and CD19-PE-Cy5 (DAKO, Denmark), CD40-PE (Becton–Dickinson, USA) - for B cells. Then intracellular labeling of CCS member proteins was performed with anti: μ-calpain, m-calpain and calpastatin (all GeneTex, USA) after fixation and permeabilisation procedure with 2% paraformaldehyde buffer and 0.25% saponin (Sigma Aldrich, Germany) in PBS. All three primary anti-CCS antibodies were conjugated with Mix-n-Stain CF555 antibody labeling kit (Biotium, Inc., USA) yielding the conjugate fluorescence identical with that of FITC. This technique produces antibodies that are uniformly conjugated to the same fluorochrome, thus allowing for semi-quantitative analysis and interpretation of the resulting fluorescence values. Determination of the net values of the mean fluorescence intensities (MFIs) of the CCS protein-bound antibodies was done using fluorescence-minus-one (FMO) controls. This technique allowed to determine the ‘negative’ MFIs (from samples stained with all conjugated antibodies minus the CF555-conjugated anti-CCS ones) to be subtracted from the relevant ‘total’ MFIs of the samples stained with all antibodies including the CF555-conjugated anti-CCS).

Negative control for surface phenotype staining was achieved by staining of parallel samples with: isotype-PE, isotype-PE-Cy5 (both IgG1), isotype-PE (IgG2b), and isotype-PE-CF594 (IgG2a) (all Becton–Dickinson, USA).

All measurements were made with FACScalibur Flow Cytometer (Becton–Dickinson, San Jose, CA, USA); at least 10^4^ cells were acquired from each sample using the CellQuest™ software of the instrument and then analyzed using the Cyflogic™ v. 1.2.1 software. At analysis, the lymphocytes were first identified by forward and side scatter gating and then by CD4, CD8, CD19 expression, respectively.

All statistical analyses were performed with STATISTICA 10™ using either the parametric *t* tests to compare two independent groups (relative to the groups or to the variable) after confirming the normality of data distribution or Mann–Whitney *U* test for data deviating from normal distribution. Statistical significance was expressed as p<0.05, as shown in the figures. All values are expressed as mean± standard deviation (SD).

## Results

**Figure 1:**
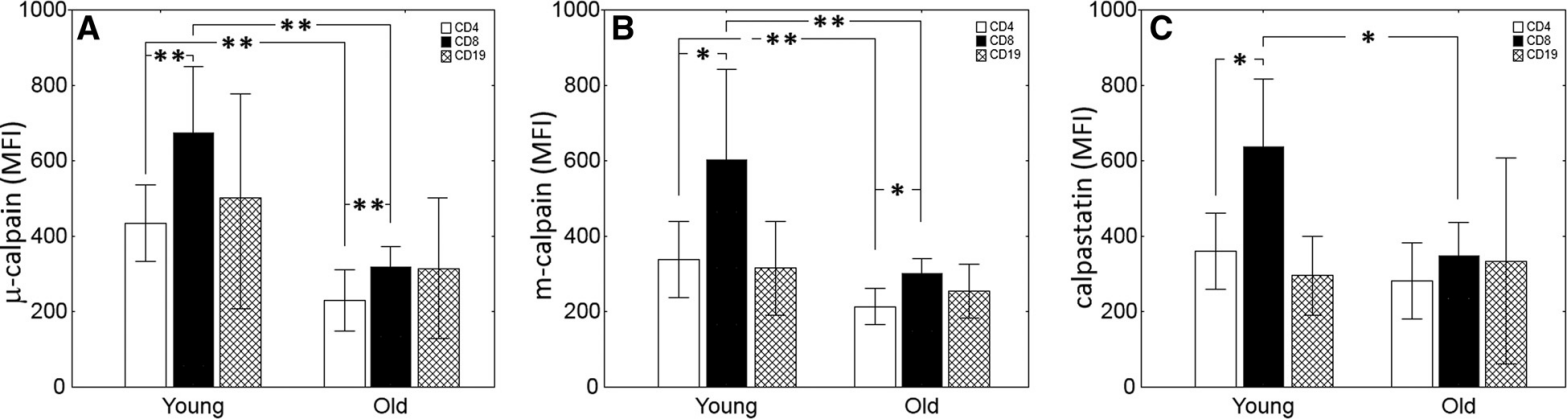
**Significant differences in the amounts of μ- (A) and m-calpain (B) and of calpastatin (C) between various populations of peripheral blood T, but not B lymphocytes (white bars - CD4**^**+**^**, black bars - CD8**^**+ **^**and patterned bars - CD19**^**+**^**) of young and old subjects.** Results are shown as bars for means and whiskers for +/-SD. Statistical significance is indicated by asterisks at p<0.05 (*) or p<0.001 (**). See Methods for the details.

Despite relatively small group of tested individuals (altogether just 10 young and 16 old people), we were already able to demonstrate highly significant (p<0.001) decreases in the amounts of both μ- and m-calpain in peripheral blood T (CD4^+^, CD8^+^) lymphocytes of old individuals as compared to young people (Figure 1 A, B). Interestingly, significant differences in the amount of both μ- and m-calpain could be seen between the CD4^+^ and CD8^+^ lymphocytes of both young and old individuals, with the respective amount being significantly higher for the CD8^+^ cells (Figure 1 A, B). Weaker, but still significant (p<0.05) differences were also found for calpastatin levels, showing a decrease in the CD8^+^ lymphocytes of elderly as compared to young subjects, as well as a significantly higher amount of this inhibitory protein in the CD8^+^ than in CD4^+^ lymphocytes of young people (Figure 1C).

**Figure 2:**
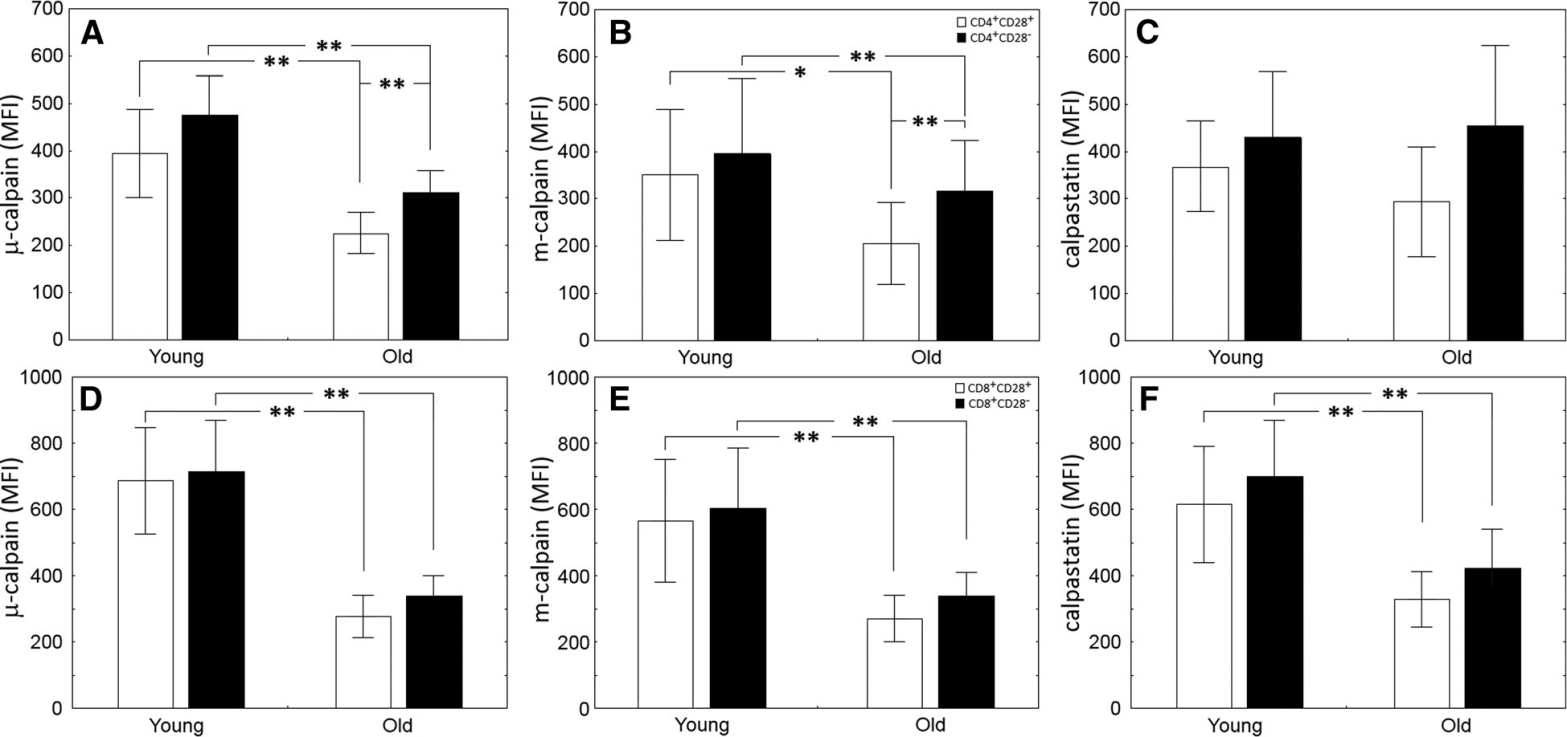
**Significantly decreased amounts of both μ- (A,D) and m-calpain (B,E) in the CD4**^**+ **^**(A,B) and CD8**^**+ **^**(D,E) as well as of calpastatin (only in the CD8**^**+**^**; C,F) lymphocytes of old people regardless the expression or lack of CD28.** Significantly higher amounts of both calpains in the CD4^+^CD28^-^ vs. CD4^+^CD28^+^ of old people **(A,B)** but not in CD8^+^CD28^-^ vs. CD8^+^CD28^+^ **(D,E)** lymphocytes of young as well as old subjects. Results are shown as bars for means (CD28^+^ (□) and CD28^-^ (■) cells respectively) and whiskers for +/- SD. Statistical significance is indicated by asterisks at p<0.05(*) or p<0.001 (**). See Methods for the details.

It is well known that with advanced age the proportions of T lymphocytes expressing the CD28 costimulatory molecule significantly drop [18]. Thus, observed differences between the expression of CCS member proteins in young and elderly subjects could be the result of decreased proportion of both CD4^+^CD28^+^ and CD8^+^CD28^+^ cells (and simultaneous rise in their CD28^-^ counterparts). For the elderly, the median proportion of CD4^+^CD28^-^ lymphocytes was 7.64% (interquartile range 5.57 – 26.22 percent), while corresponding values for the young subjects were 3.38% (1.48 – 6.04%) respectively. Despite strong and expected trend towards higher percentage of CD28^-^-ve population among the CD4^+^ lymphocytes in the elderly, the difference had not reached statistical significance, possibly due to high variability of the results in the elderly group (Mann–Whitney *U* test, p=0.078). Similar strong, but not significant (p=0.261) trend was observed when the proportions of CD8^+^CD28^-^ lymphocytes were compared. For the elderly, the median proportion of this population was 66.59% (25.02 – 72.14%), while for the young subjects it was 29.4 (19.29 – 46.26) %. Therefore, we have compared the relative levels of expression of the three CCS member proteins separately in CD28^+^ and CD28^-^ subpopulations of CD4^+^ and CD8^+^ lymphocytes (Figure 2). Here, the most striking observations concern significantly higher expression of μ-and m-calpain in the CD4^+^CD28^-^ then in the CD4^+^CD28^+^ lymphocytes of old individuals (Figure 2A, B). There are no such differences if the T cells of young individuals are considered. Interestingly, significantly lower expression of both calpains in the CD4^+^ and CD8^+^ cells of elderly subjects shown in the Figure 1 is preserved in both subpopulations of these cells differing in CD28 expression; thus, regardless of being CD28^+^ or CD28^-^, the lymphocytes of both lineages of the elderly express significantly less μ- and m-calpain than those derived from young individuals (Figure 2A, B, D, E). Weakly significant drop in the amount of calpastatin could only be demonstrated when the levels of this inhibitor were compared between CD8^+^CD28^+^ and CD8^+^CD28^-^, but not between CD4^+^CD28^+^ and CD4^+^CD28^-^ lymphocytes of young and elderly subjects (Figure 2 C, F).

**Figure 3:**
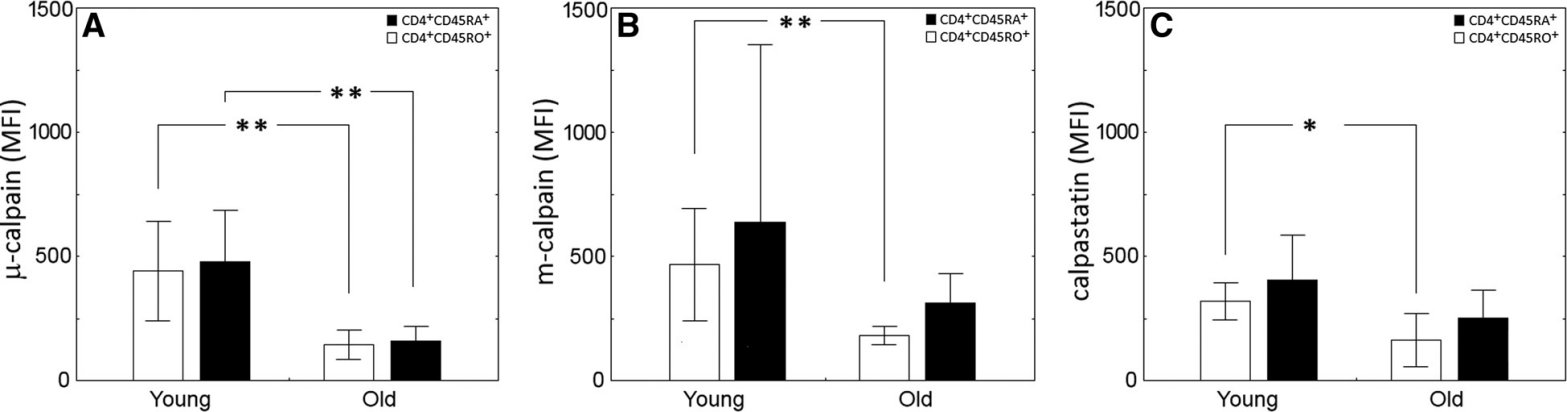
**Significantly decreased amounts of μ- and m-calpain and calpastatin in the naive (□ CD45RA**^**+**^**) and effector/memory (■ CD45RO**^**+**^**) subpopulations of CD4**^**+ **^**helper lymphocytes of old compared to young subjects, and lack of differences between these two subpopulations regarding the CCS protein amounts.** Results are shown as bars for means and whiskers for +/- SD. Statistical significance is indicated by asterisks at p<0.05(*) or p<0.001 (**). See Methods for the details.

Ageing of the adaptive immune system is also associated with the increased proportion of effector/memory (CD45RO^+^) at the expense of naïve (CD45RA^+^) lymphocytes within the CD4^+^ population [18]. Thus, it was feasible that the observed changes in the CCS expression in the CD4^+^ cells of the elderly would be due to rising proportion of the CD45RO^+^ cells. In the investigated subjects a strong, statistically significant (Mann–Whitney *U* test, p=0.0132) difference was shown when the proportions of memory CD4^+^CD45RO^+^ lymphocytes were compared. Thus, median value for this proportion for young subjects was 39.46% with the interquartile range from 32.65 – 45.25%, and for the elderly subjects it was 59.13 (49.21 – 65.07) % respectively. Therefore, we have checked if these memory cells differ from CD45RA^+^ lymphocytes with respect to the expression of any of the CCS members. As shown in the Figure 3, there were no differences in the expression of both μ- and m-calpain between the naïve and effector/memory cells, regardless the age of cell donor. The naïve (CD45RA^+^) subpopulation of the CD4^+^ cells derived from elderly exhibited significantly lower amounts of both calpains and of calpastatin than cells of young people (Figure 3 A,B,C).

## Discussion

Ageing of the adaptive immune system manifests as increased prevalence of infections, neoplasms and autoimmune diseases in the elderly and aged cohorts as compared to young and even middle-aged individuals. At the cellular level, multiple shifts in the proportions of lymphocytes exhibiting various phenotypes are observed, notably increased proportions of the effector/memory and TEMRA cells at the expense of naïve lymphocytes. Accompanying (and, presumably, causatively underlying) functional changes in the lymphocytes of elderly people include overall lower efficiency of proliferation to antigen and mitogen challenges in vitro, decreased production of multiple cytokines and increased proportions of lymphocytes undergoing activation-dependent apoptosis (AICD) [18,19]. We have shown before, that the dynamics of the cell cycle in the CD4^+^ lymphocytes of aged people exhibits significant elongation of the time between onset of stimulation and beginning of the first G1 phase of the first division cycle (the G0→G1 time) in a manner dependent on the level of costimulatory CD28 molecule on the surface of these cells, as well as shortening of the G1 phases of the ensuing division cycles, associated with increased amounts of cyclin D1 bound to the cyclin-dependent kinases (i.e. active) [19].

The calpain-calpastatin system (CCS), consisting of two ubiquitous cellular proteases (μ- and m-calpain) and their endogenous inhibitor – calpastatin [1-5] is commonly acknowledged to variably regulate multiple cellular activities, including proliferation and apoptosis, by token of calpains’ ability to modulate the activities of the involved proteins by cleaving only their regulatory domains [1-10]. One of the important substrates of calpains is cyclin D1, a member of cyclin D family deciding the timing and progression of the G1 phase of the cell cycle [20]. Thus it was feasible, that the activity of CCS would be modified in the immune cells of healthy elderly. Both ubiquitous calpains and calpastatin are universally expressed in human T lymphocytes (there are no ‘calpain-negative’ T cells [15]) and their functions in these cells are involved in modifying the differentiation of Th1, Th2 and Th17 cells [21] and in NF-kB-dependent activation [22], as well as in the regulation of the level of ZAP70 [23]. We have shown before a strong, ageing-dependent decrease in the available activity of μ-calpain assessed by casein zymography in the lysates of total (unseparated) peripheral blood lymphocytes [14]. However, to date only that one and our another paper on age-dependent changes in the CD4^+^ lymphocyte cell cycle dynamics cited above [19] as well as a paper from another group indirectly suggesting the involvement of calpain activity in the B cell precursor senescence in mice [24] have been published on the topic of calpain involvement in the lymphocyte ageing.

In this preliminary paper we attempted to compare the expression of μ- and m-calpain and calpastatin (forming the CCS system of limited regulatory proteolysis) in the major populations of human peripheral blood lymphocytes derived from healthy young and elderly Sicilian and Polish subjects, as a pilot study for an international, Polish-Italian project CALPACENT, aiming at qualitative, quantitative and functional comparison/assessment of the CCS in centenarian versus the general elderly and younger cohorts. Despite the relatively limited numbers of individuals tested so far, we were able to show that all three member proteins of the CCS are detectable and relatively quantifiable by flow cytometry, and that their expression is significantly lower in the T cells of elderly subjects, regardless their phenotypic characteristics. Thus we can conclude that ageing-associated decrease in the amounts of CCS member proteins in the peripheral blood T (but not B) lymphocytes is a universal phenomenon. This decrease was the most prominent for the CD8^+^ lymphocytes; on average, the decrease of the amount was by 60% for μ-calpain, and by 50% for m-calpain and calpastatin, where the same figures for CD4^+^ cells were 45%, 40% and only 20% respectively. These figures show the trend towards a major difference; however, they did not reach statistical significance, probably due to too small numbers of subjects studied.

The latter finding may be of potential significance (drop in calpain without or with only a slight simultaneous drop in calpastatin levels in the CD4^+^ cells shifts the stoichiometry within the CCS, making possible loss of total and momentous calpain activities due to relative excess of the endogenous inhibition); it was recently shown that calpastatin overexpression suppresses production of IL-6 and development of Th17 lymphocytes and thus is anti-inflammatory [21]. Reduction of calpains’ expression with no change in calpastatin expression should have similar effect, thus possibly acting as a homeostatic countermeasure against increased inflammatory readiness in the aged individuals (inflamm-ageing).

As ageing of the immune system is associated with an increase in the proportion of CD4^+^CD28^-^ cells at the expense of CD4^+^CD28^+^ lymphocytes on one side, and with rising proportions of memory CD45RO^+^ lymphocytes at the expense of naïve CD45RA^+^ ones, it was conceivable that the differences in the amounts of CCS proteins demonstrated between young and elderly subjects would stem from changed proportions of the abovementioned subpopulations. However, the only significant difference concerning the CCS protein amounts between major age-dependent differences in the cellular abundance of member proteins of the CCS could be shown for μ- and m-calpain between the CD28^+^ versus CD28^-^ within the CD4^+^ population of old people; no such difference could be seen when lymphocytes differing in CD28 expression was compared for young individuals, nor within the CD8^+^ population.

These observations mean that the rate of ageing-associated decrease in the calpain amounts in the CD4^+^CD28^+^ lymphocyte population is higher than in the CD4^+^CD28^-^ counterparts. The CD4^+^CD28^-^ lymphocytes are suggested to acquire their phenotype in relation to increased proinflammatory state of the individual (notably increased levels of TNF [25]), and themselves participate in inflammatory processes including rheumatoid arthritis, chronic kidney disease and atherogenesis [26-29]. It was suggested that TNF augments the expression and activity of calpains and that its proinflammatory activity is partially dependent on calpain activation [30]. Thus, relatively retained levels of calpains in CD4^+^CD28^-^ cells of the aged (as compared to these levels in the CD4^+^CD28^+^ cells) may be a consequence of higher response of the former to TNF following the concept of inflamm-ageing [31]. Why such differences could not be demonstrated for the CD8^+^CD28^+^ and CD8^+^CD28^-^ cells of the elderly (showing uniform drop in CCS proteins’ expression with advanced age) remains a mystery; however one is tempted to speculate, that the appearance of CD8^+^CD28^-^ lymphocytes is relatively less dependent on the activity of TNF and that the reaction of the CCS system follows suit.

On the other hand, the expected (and duly observed here), significant age-related increase in the proportion of CD4^+^CD45RO^+^ memory lymphocytes is of no consequence for the overall decrease in the amount of calpains, as we did not show any difference in the enzymes’ amounts between the naïve and memory lymphocytes.

The important question remaining is what is the physiological consequence of the described changes in the amounts of calpains and calpastatin in the elderly lymphocytes, and whether they might be cause or rather effect of these cells entering immunosenescence. Based on our early paper we can conclude that decreased amount of the enzymes directly corresponds to their decreased available activity [14]. This notion is corroborated by our observations of increased levels of their important endogenous substrate – the D1 cyclins [20] - in the CD4^+^ lymphocytes of the elderly, correlated with significantly shorter time required by these cells to complete the mitotic cycle [19]. It was also suggested, that retention of high levels of D1 cyclin prevents cells from entering the S phase, reducing their proliferation [32], which may be the case for elderly T cells exhibiting reduced CCS. On the other hand, calpain activity is known to be conductive for either increased apoptosis or apoptosis escape of different cell types [7-10,14]. It is well known that the CD4^+^ cells of the elderly are relatively resistant to apoptosis, which could be interpreted as at least in part dependent of decreased pro-apoptotic activity of lower amounts of calpains seen in these cells.

A necessary precondition of intracellular activation of calpains is the elevation of cytoplasmatic ionized calcium level above its resting values. It is well known that this ‘calcium signal’ is decreased in the stimulated T cells and other leukocytes of old individuals [33,34]. This decrease may result in lower ‘attainable’ activity of μ- and especially m-calpain (the latter requiring 100 micro- to 1 millimolar Ca^2+^ for its activation in vitro, and similar calcium concentration in vivo [1-3,35]) in activated T cells of old individuals, in turn leading to decrease of (activation-dependent) apoptosis of these cells. The latter paper points at an interesting effect of m-calpain activity in the T cells, i.e. specific participation in the movements (migration) of these cells [35]. It is thus possible, that lack of calpain (and its activity) in the T cells of old individuals may impair migration, and in consequence tissue infiltration by these cells upon activation.

What would be the reason for lower amounts of the CCS member proteins in the T cells of elderly? The most obvious seems to be the decreased transcriptional activity of the relevant genes. However, in a preliminary experiment we were unable to show that this decrease in calpain protein amount and activity is related to decreased level of the calpain genes’ (CANP) transcription. Also, we did not see any age-dependent change in CANP transcription levels in non-transformed human lymphocytes in the previous paper [14]. On the other hand, the activity of calpains is self-limiting, in the sense that activated calpain is its own substrate; thus, intracellular activation of calpain would lead in time to the reduction of its amount [3,10], which we in fact observed for the lymphocytes of elderly. This explanation would, however, require some prior level of endogenous activity of calpains; again, in a preliminary experiment using the technique we described earlier for measuring endogenous activity of calpains ex vivo as specific calpastatin degradation [15]) we did see hallmarks of calpain activity (as endogenous calpastatin degradation) in the T cells of old, but not young people. Support to the hypothesis of endogenous activation of calpains in the T cells of elderly, possibly leading to decreased amounts of the enzyme that we observe in these cells, is lent by observation of increased “resting” Ca^2+^ in both T cells and neutrophils of elderly, interpreted by these authors as ‘priming’ (i.e., early stage of activation) of these cells [33,34].

## Conclusions

Our findings suggest that the decrease of CCS (μ- and m-calpain and calpastatin) expression in the T lymphocytes with advanced age is universal but more pronounced for the CD8^+^ lymphocytes, possibly affecting their function in the aged. Further studies are required to elucidate both the mechanism of the observed decreases in CCS expression and its role in impaired function of old T lymphocytes. We believe that these preliminary findings lend momentum to the more detailed study of the relation between CCS expression and activities in the lymphocytes and human longevity.

## Competing interests

The authors declare that they have no competing interests.

## Authors’ contributions

AM performed cytometric experiments (Poland, Sicily), prepared material for molecular studies, analysed data and drafted the manuscript. JF recruited subjects for all age cohorts (Poland), provided detailed clinical characteristics w/Senieur protocol, provided the biological material, participated in drafting the manuscript. AJ performed cytometric experiments (Poland), analysed data and participated in drafting the manuscript. JF performed cytometric experiments (Poland), prepared material for molecular studies, analysed data and participated in drafting the manuscript. GC-R coordinated the recruitment of Sicilian subjects and laboratory studies, and participated in drafting the manuscript. MB performed cytometric experiments (Sicily) and participated in drafting the manuscript. SB performed cytometric experiments (Sicily) and participated in drafting the manuscript. AM performed cytometric experiments (Sicily) and participated in drafting the manuscript. CC coordinated the recruitment of Sicilian subjects and Sicilian part of the project-related experiments; participated in drafting the manuscript. EB analysed data and participated in drafting the manuscript. JMW authored the project hypothesis and aims, coordinated the Sicilian and Polish studies, analysed data, formulated the conclusions, authored the final version of the manuscript. All authors read and approved the final manuscript.
